# Modified Single–Working Portal Technique Using Percutaneous Spinal Needle Suture Passing in Arthroscopic Subscapularis Repair

**DOI:** 10.1016/j.eats.2023.102898

**Published:** 2024-01-16

**Authors:** Hong Wang, Wenbo Yang, Chunqing Meng, Shuyan Wu, Wei Yu, Wei Huang

**Affiliations:** Department of Orthopaedics, Union Hospital, Tongji Medical College, Huazhong University of Science and Technology, Wuhan, China

## Abstract

The main method for arthroscopic repair of the subscapularis is repair with suture anchors. The surgeon generally establishes the anterior and anterolateral operation portals to complete anchor implantation and suture passing, respectively. The single–operation portal technique has been developed recently. However, in the traditional single–operation portal technique, the suture device and grasper are difficult to operate simultaneously. In addition, with the traditional rotator cuff suture device, it is easy to cause further iatrogenic injury to the rotator cuff because of its larger diameter. Therefore, we describe a modified single–operation portal technique for suture passing percutaneously with a spinal needle taking into account the shortcomings of existing techniques. Our modified technique avoids the use of traditional suturing devices and effectively avoids further damage to the rotator cuff. The use of a single operation portal makes the operation more minimally invasive and simple and effectively avoids the problem of interference between the suture device and grasper in the same portal. The entire operational process does not require the use of costly consumables, resulting in increased cost-effectiveness and a significantly reduced operating time. In conclusion, our modified technique achieves the use of a single operation portal to suture the subscapularis through spinal needle suture passing, which has good clinical value.

The subscapularis plays an important role in shoulder joint activities as the only internal rotation muscle in the body. Subscapularis injury typically involves a partial tear in the superior third of the tendon, causing pain and loss of mobility of the shoulder joint. The most common treatment for subscapularis tears is arthroscopic repair with suture anchors, with good postoperative results. Arthroscopic subscapularis repair was proposed by Burkhart and Tehrany^1^ in 2002 and has been developed in recent years,^2^ generally using 2 operation portals. Recently, a single–working portal technique has been proposed,^3^ which is more minimally invasive and simple. However, suturing and thread catching need to be performed sequentially in a single portal, and this may cause suture management problems. There are many suturing tools for subscapularis suturing, including suture hooks, suture passers, and penetrators, that will inevitably cause iatrogenic damage to tendon tissue, especially penetrators with relatively thick diameters. The use of a lumbar needle for rotator cuff suturing can effectively reduce the additional damage caused by traditional rotator cuff suture devices.^4^ We present a modified single–working portal technique using percutaneous spinal needle suture passing in arthroscopic subscapularis repair. This technique makes the surgical procedure simpler, more convenient, and minimally invasive and avoids the interference of sutures.

## Surgical Technique

Our surgical protocol is shown in Video 1. The main steps are described in the following sections.

### Patient Preparation and Portal Establishment

**Fig 1:**
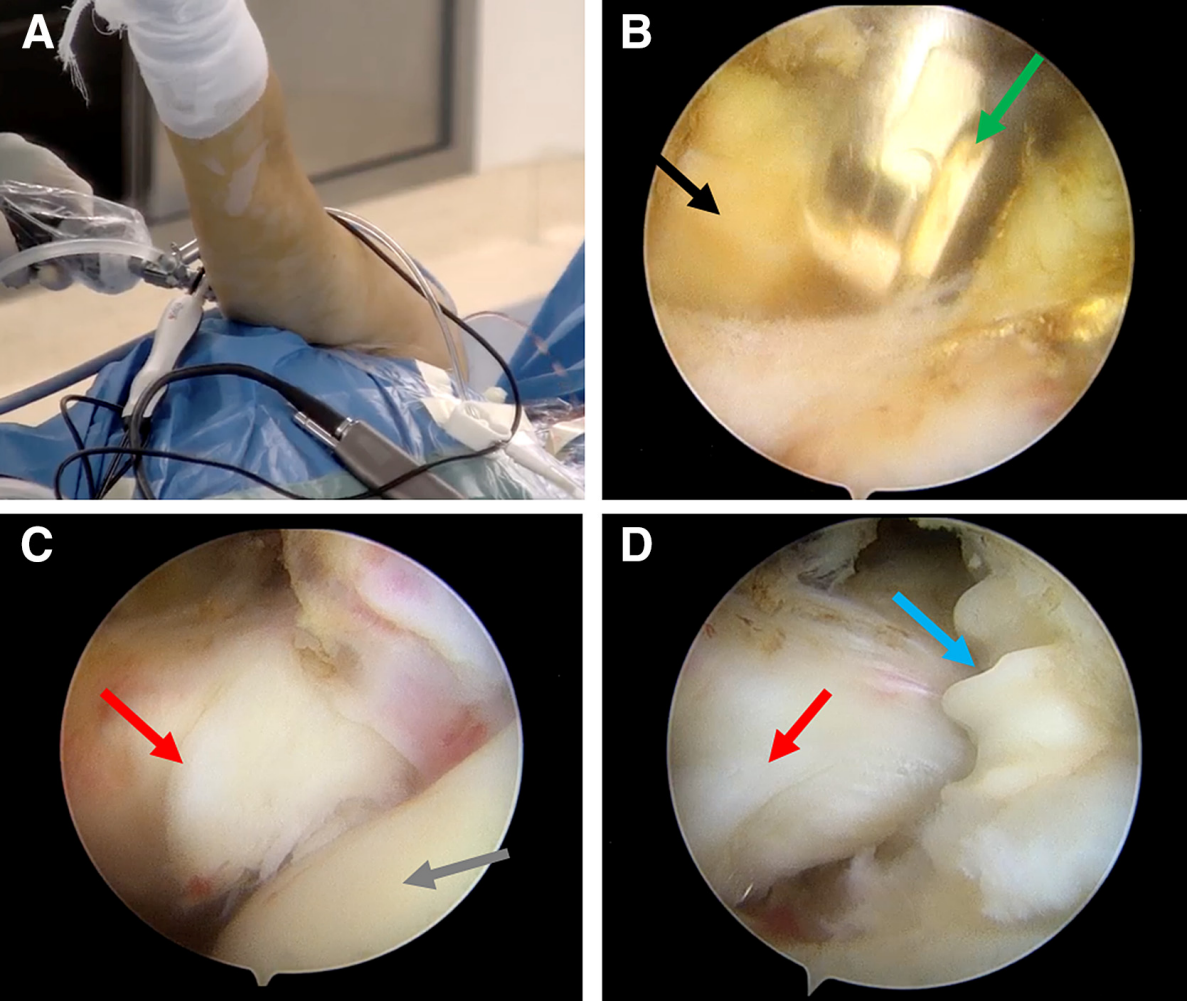
Surgical position, tear site exposure, freshening and suture anchor implantation. (A) In this example, the patient with affected right shoulder is placed in a lateral surgical position. (B) The radiofrequency is used to clean the rotator cuff interval though the anterior operation portal. (C) Superior third subscapularis tear is visible in the right shoulder through the posterior observation portal. (D) The state of the implanted anchor. In this figure, the red arrow indicates the tear. The green arrow indicates the rotator cuff interval. The black arrow indicates the radiofrequency. The blue arrow indicates the anchor.

After receiving general anesthesia, the patient is placed in a lateral position with the surgical arm retracted and a 5-kg weight is suspended, followed by routine disinfection and draping. The right shoulder position is 50° of abduction and 15° of forward flexion (Fig 1A). First, a conventional posterior portal is created as an observation portal, and a 30° arthroscope is used to observe the glenohumeral joint. Then, the anterior operation portal is created, and a radiofrequency device is used to clean the rotator cuff interval (Fig 1B).

### Tear Detection and Suture Anchor Implantation

A tear of the superior third of the subscapularis is detected from the posterior observation portal (Fig 1C). Freshening is performed with a shaver after the bone bed of the footprint area of the subscapularis is exposed. Then, a double-loaded anchor (Fastlock PK; Star Sports Medicine, Beijing, China) is implanted at the footprint of the subscapularis (Fig 1D).

### Spinal Needle Preparation and First Percutaneous Suture Passing

**Fig 2:**
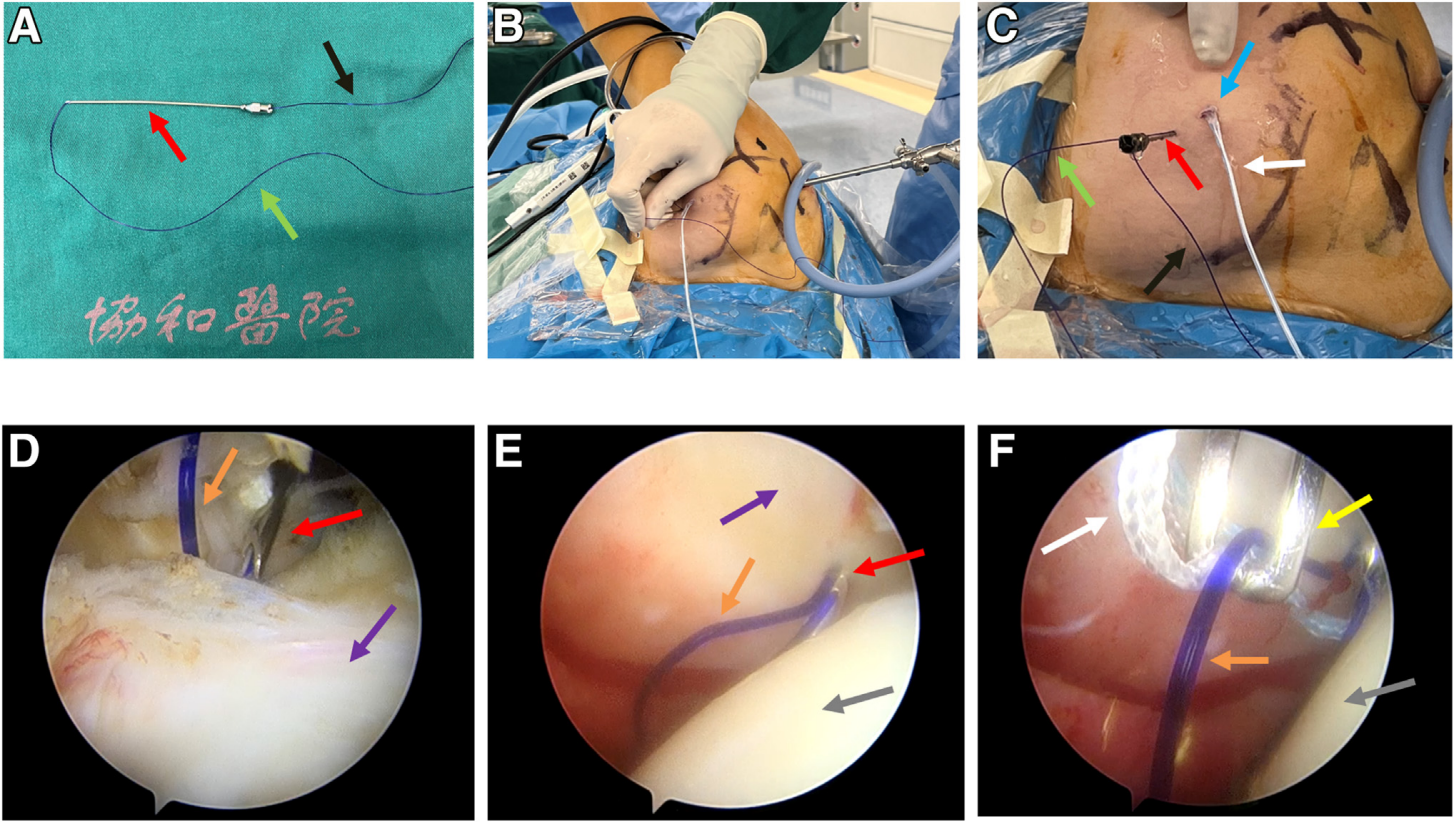
Spinal needle preparation and process of percutaneous spinal needle suture passing for first time. The posterior portal is used as the observation portal in a right shoulder. (A) A 12-gauge spinal needle (red arrow) loaded with traction PDS suture (black arrow) is prepared. Half of the PDS suture is left outside the needle, as indicated by the green arrow. (B, C) The spinal needle loaded with traction suture is inserted percutaneously below the anterior portal (blue arrow) to penetrate the subscapularis tendon: in vitro view. The white arrow indicates the anchor suture. (D) The spinal needle loaded with traction suture is inserted percutaneously below the anterior portal to penetrate the subscapularis tendon: in vivo view. The spinal needle is percutaneous and reach to the rotate cuff rotator interval before penetrating the tendon. The orange arrow indicates the traction suture loop structure; the purple arrow, the subscapularis tendon. (E) Spinal needle after penetrating tendon. The gray arrow indicates the humeral head. (F) The traction suture loop and one of the white anchor sutures are pulled out of the joint through the anterior portal with the grasper (yellow arrow).

A 12-gauge spinal needle loaded with traction suture (PDS II; Ethicon, Somerville, NJ) is prepared; half of the PDS suture is left outside the needle (Fig 2A). Then, we insert the spinal needle percutaneously below the anterior portal to penetrate the subscapularis tendon (Fig 2 B-E). The traction suture loop and one of the white anchor sutures are pulled out of the joint through the anterior portal with a grasper (Fig 2F). Then, the white anchor suture is percutaneously passed out with the traction suture loop.

### Second Percutaneous Suture Passing

**Fig 3:**
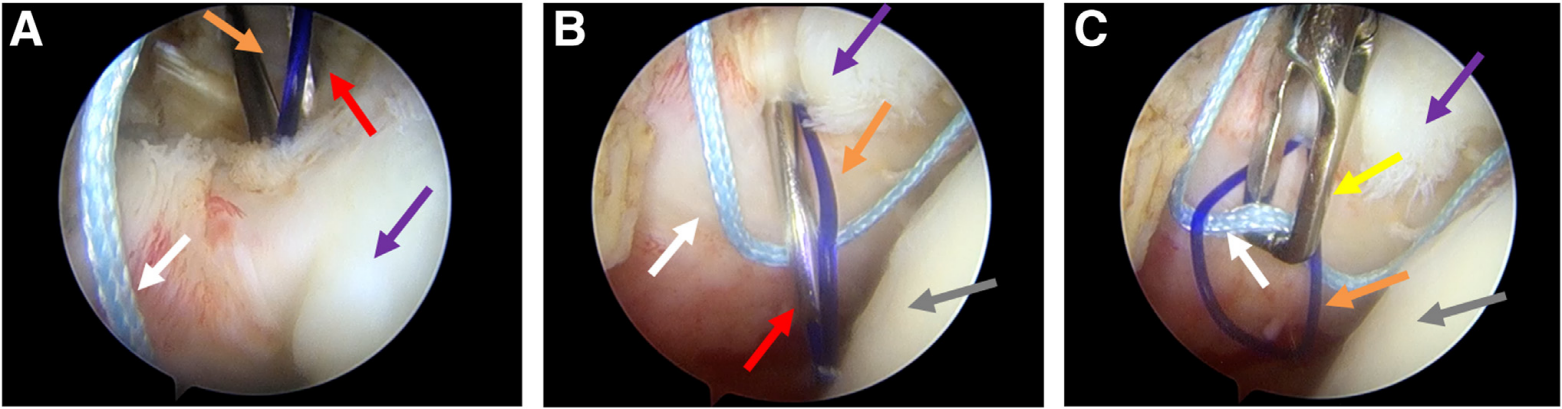
Second percutaneous suture passing. The posterior portal acts as the observation portal in a right shoulder. (A) Spinal needle (red arrow) before penetrating tendon. The orange arrow indicates the traction suture loop structure; the purple arrow, the subscapularis tendon; and the white arrow, one of the blue anchor sutures. (B) Spinal needle after penetrating tendon. The gray arrow indicates the humeral head. (C) Process of suture passing in joint. The grasper (yellow arrow) enters through the anterior portal, pulls out of a blue anchor suture.

The second suturing of the subscapularis is performed in the same way. The spinal needle is percutaneously punctured into the rotator cuff interval at another position below the anterior portal (separate from the white anchor suture position), and it then passes through the upper part of the subscapularis tendon above the white anchor suture (Fig 3 A and B). Different from the first suture passing, the grasper enters through the anterior portal, pulls out of the blue anchor suture after the suture passing through the traction suture loop in the joint (Fig 3C). Then, the blue anchor suture is percutaneously passed out with the traction suture loop.

### Knotting and Fixation

**Fig 4:**
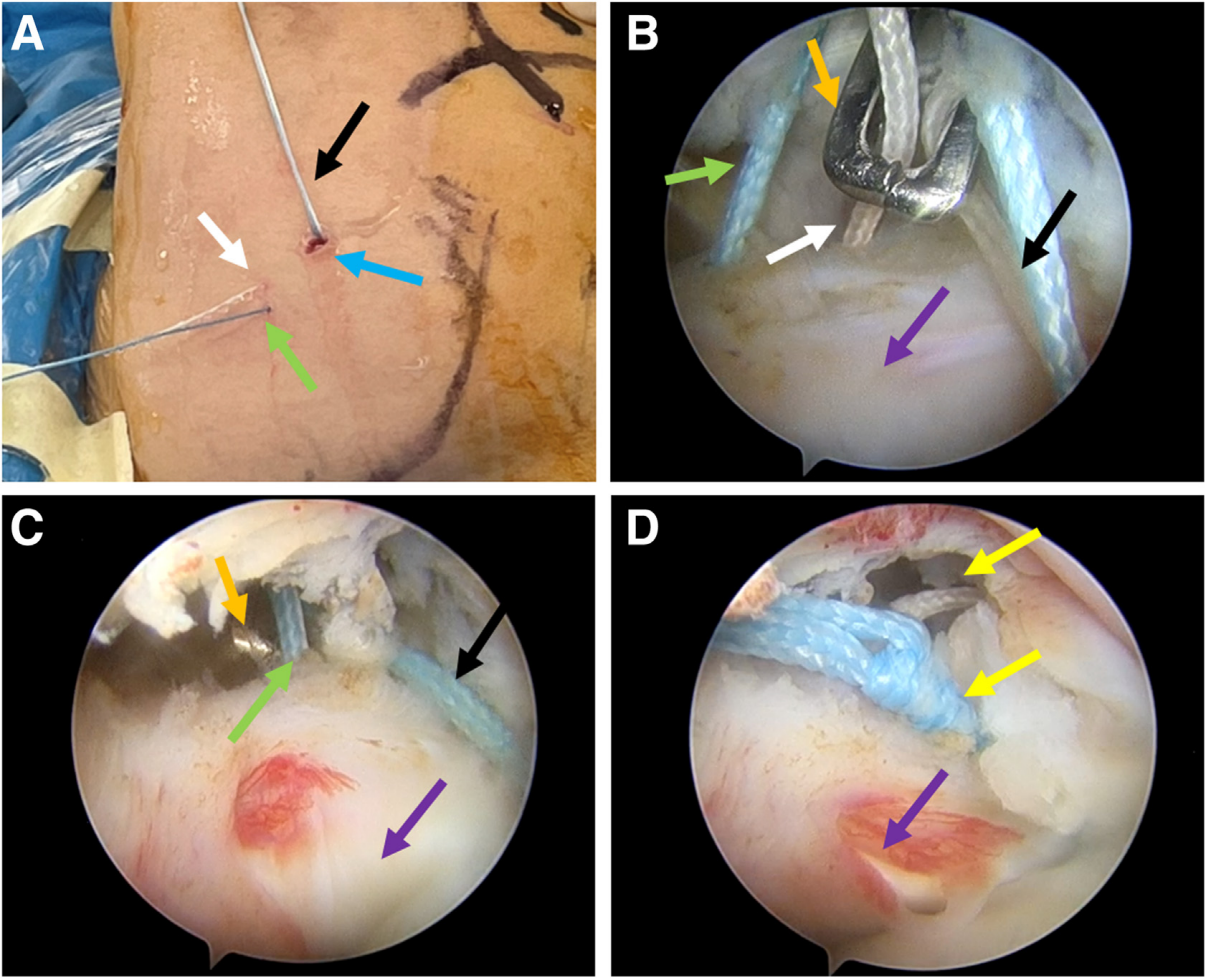
Knotting and fixation. The posterior portal acts as the observation portal in a right shoulder. (A) Anchor suture distribution after percutaneous suture shuttling: in vitro view. The white arrow indicates the white anchor suture penetrating the inferior part of the subscapularis; the green arrow, the blue anchor suture penetrating the superior part of the subscapularis; the black arrow, the 2 anchor sutures without penetrating the tendon; and the blue arrow, the anterior portal. (B) The inferior white sutures are pulled out through the anterior portal with the grasper (orange arrow) and knotted first. The purple arrow indicates the subscapularis tendon. (C) The superior blue anchor sutures are pulled out through the anterior portal with the grasper and knotted. (D) Arthroscopic observation after subscapularis repair. The yellow arrows indicate 2 knots.

As Figure 4 shows, 2 anchor sutures are placed in the anterior portal without penetrating the tendon, and the 2 penetrated sutures are passed out percutaneously. The 2 white anchor sutures (lower sutures) are pulled out with the grasper through the anterior portal and knotted first; the blue anchor sutures follow the same process.

## Discussion

Subscapularis injury is a frequently encountered condition affecting the shoulder muscles, which can potentially impair the range of motion in the shoulder joint. Currently, arthroscopy serves as the primary technique for repairing subscapularis injuries.^5^ The technique of total arthroscopic subscapularis repair mainly refers to repair through suture anchors, including single- and double-row suture anchors.^6^ In the course of surgery, 2 operation portals are generally adopted, consisting of the anterior portal and the anterolateral portal. The anterior portal is primarily used for anchor placement, whereas the anterolateral portal is used for other operations (including freshening of the anchor placement area and suture passing). In recent years, a single-portal technique for subscapularis repair has been proposed, which is convenient to perform, can save operating time, and yields less damage to the patient. However, there are some problems with the single-portal technique, such as the inability of the suture device and grasper to operate within the joint at the same time, as well as suture management issues with the single portal. Moreover, in the traditional single-portal technique, the positioning of the portal is very important. This single operation portal is to be used both for suture anchor implantation and for suture device operations. The experience and skill of clinicians are thus subject to stringent requirements. Our improved technique allows for greater flexibility in the choice of location for operation portal establishment, given that the spinal needle used for the suture is passed through the skin to the injury site. The only operation portal is used primarily for anchor implantation, grasper operation, and suture knotting, without the need for a suture device. In addition, iatrogenic tendon injury caused by suture devices cannot be ignored, whether performing traditional tendon suturing with 2 operation portals or performing the single-portal technique. There are many kinds of suture devices used for rotator cuff suturing, both forward suturing devices and reverse suturing devices, such as penetrators, suture passers, and suture hooks.^7^^,^^8^ These devices can cause iatrogenic damage to tendon tissue because they have large diameters. In theory, the ideal suture tool should have the smallest possible diameter. The diameter of the 12-gauge spinal needle used in our technique is much smaller than that of existing suture equipment and thus allows for minimal tendon damage.

Overall, the main feature of our technique is a modified single operation portal to repair the subscapularis through suture passing with a percutaneous spinal needle. Compared with the traditional single–operation portal technique, our technique is less invasive to patients, is more convenient and economical, and is suitable for beginners to learn. In addition, the equipment used in our technique is common in the operating room, and it is easy for surgeons to find the required equipment or related replacable equipment, which has strong promotion significance. In our technique, because the spinal needle used for suturing is percutaneous to reach the lesion site, it is not limited to suturing the lower part of the subscapularis. We summarize the pearls and pitfalls of our technique in Table 1 and the advantages and disadvantages in Table 2. In conclusion, our technique has good application value in arthroscopic repair of the subscapularis.

**Table 1:** Pearls and Pitfalls of Modified Single–Working Portal Technique Using Percutaneous Spinal Needle Suture Passing

Pearls
The spinal needle selected should be as small as possible to reduce iatrogenic injury to tendon tissue. At the same time, the spinal needle must not be easy to bend, and its diameter should be able to pass through the traction suture.
The establishment of the anterior portal is mainly based on the need to implant the suture anchor.
The percutaneous position of the spinal needle is usually below the anterior portal, mainly to facilitate reaching the subscapularis. If the puncture angle is not good, the spinal needle can be pulled out and the appropriate percutaneous puncture point can be selected again.
The rotator cuff interval should be cleared with a radiofrequency device prior to puncture, especially around the subscapularis, so as to better reveal the puncture point of the spinal needle in front of the subscapularis.
Suture passing is performed through a loop formed by a spinal needle loaded with traction suture. There are 2 types of suture passing: intra-joint suture passing and out-of-joint suture passing. We believe that intra-joint suture passing is more simple, convenient, and safe and can be completed independently without relying on assistants.
Once the suturing is finished, the suture that has gone through the tissue and the suture that has not gone through the tissue should be pulled out through the anterior operation portal with a grasper before tying. We recommend that the suture located on the lower subscapularis be knotted first.
Pitfalls
The spinal needle should not be too medial during percutaneous puncture to avoid damage to blood vessels and nerves.
The rotator cuff interval should be cleaned as much as possible to avoid problems with suture management.

**Table 2:** Advantages and Disadvantages of Modified Single–Working Portal Technique Using Percutaneous Spinal Needle Suture Passing

Advantages
The spinal needle has a smaller diameter than traditional suture hooks, resulting in less iatrogenic damage to rotator cuff tissue.
The technique only requires the use of 1 operation portal to carry out suture anchor implantation, suture passing, and knotting, thus achieving a more minimally invasive and simple model.
The suturing is completed by percutaneous puncture, effectively avoiding interference between the suture device and grasper in the same portal, as well as facilitating suture management.
The anterior portal in this technique is created with greater flexibility in location, and the technique is easy for beginners to learn.
The technique uses a spinal needle to load the PDS suture into a large loop structure that accommodates the passage of a grasper and can be completed directly in the joint by a single surgeon.
The tools used in the technique are highly fungible, such as hollow needles.
The whole process is simple, economical, and convenient, and the operating time is short.
The spinal needle is straight, and the process of penetrating the tissue is more straightforward.
The technique is suitable for tears of the inferior part of the subscapularis and tear of the subscapularis with retraction.
Disadvantages
Percutaneous suturing is a demanding technique when performed arthroscopically and requires a learning curve.
The tissue may be at risk of damage during percutaneous puncture; therefore, meticulous attention should be given to the precise location of the puncture.

## Disclosures

The authors report no conflicts of interest in the authorship and publication of this article. Full ICMJE author disclosure forms are available for this article online, as supplementary material.

## Supplementary Data

ICMJE author disclosure forms

Video 1 Introduction of modified single–working portal technique using percutaneous spinal needle suture passing in arthroscopic subscapularis repair. In the example, the patient with affected right shoulder is placed in a lateral surgical position. Once the tear has been freshened, suture anchor is placed. We then show in the video how to assist the anchor suture passing with a spinal needle.
